# Supplementary material for: Studies on Chemical IR Images of Poly(hydroxybutyrate–co–hydroxyhexanoate)/Poly(ethylene glycol) Blends and Two-Dimensional Correlation Spectroscopy
Source: Polymers (Basel). 2019 Mar 17;11(3):507. doi: 10.3390/polym11030507 (PMC6473784; doi:10.3390/polym11030507)
Supplement: Supplementary file 1 [file polymers-11-00507-s001.pdf]

polymers

Supplementary Materials

Studies on Chemical IR Images of Poly(hydroxybutyrate–*co*–hydroxyhexanoate)/ Poly(ethylene glycol) Blends and Two-Dimensional Correlation Spectroscopy

Yeonju Park[1], Sila Jin[1], Yujeong Park[1], Soo Min Kim[1], Isao Noda[2,3,*], Boknam Chae,[4] and Young Mee Jung[1,*]

[1] Department of Chemistry, Institute for Molecular Science and Fusion Technology, Kangwon National University, Chuncheon 24341, Korea; ymjung@kangwon.ac.kr
[2] Department of Materials Science and Engineering, University of Delaware, Newark, DE 19716, USA; noda@udel.edu
[3] Danimer Scientific, 140 Industrial Blvd. Bainbridge, GA 39817, USA; noda@udel.edu
[4] Pohang Accelerator Laboratory, POSTECH, Pohang 37673, Korea; cbn@postech.ac.kr
* Correspondence: noda@udel.edu (I.N.), ymjung@kangwon.ac.kr; Tel.: +82-33-250-8495 (Y.M.J.)

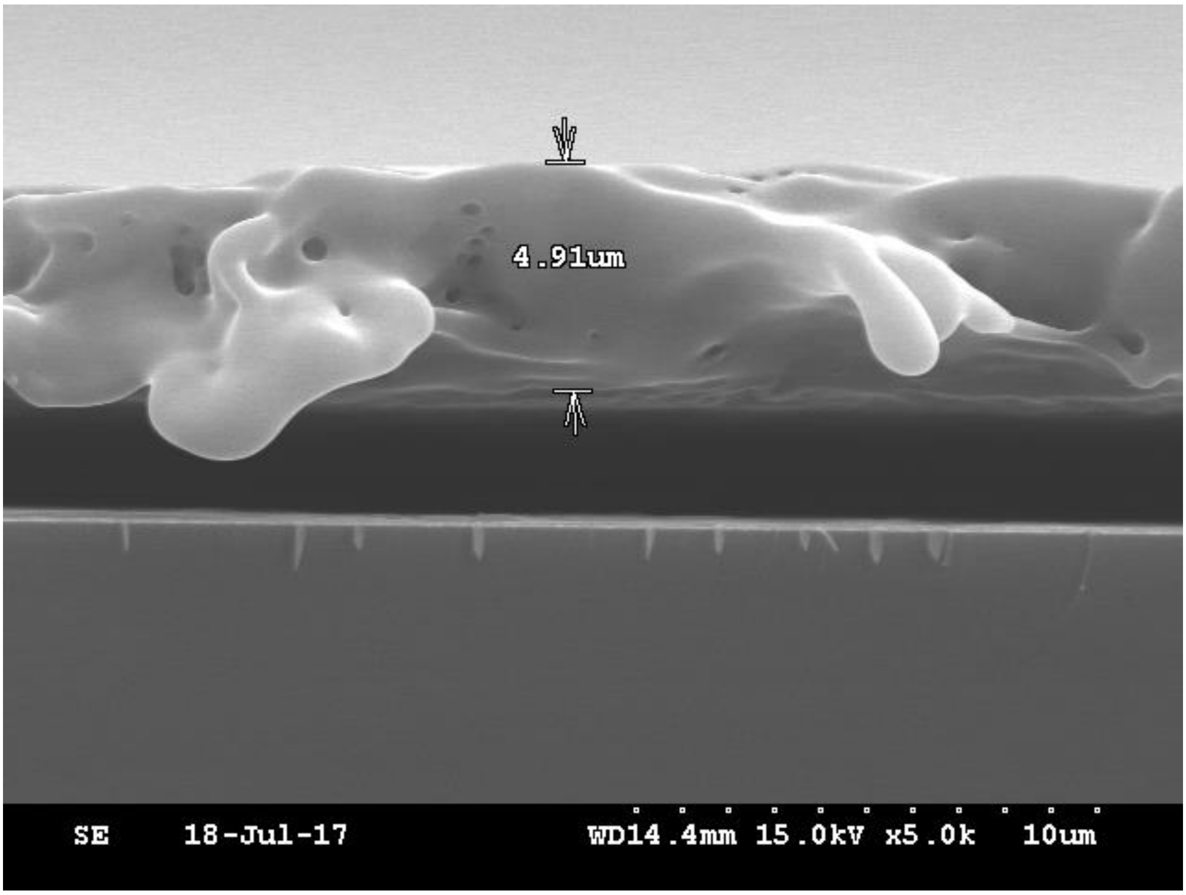

Figure S1. SEM image of cross section of spin-coated PHBHx/PEG=70/30 blend film on Pt wafer. The arrow indicates the thickness of the PHBHx/PEG=70/30 blend film.
